# A cross-sectional study on factors associated with health care seeking for acute respiratory infection and fever in children under-five in Zambia

**DOI:** 10.11604/pamj.2023.44.125.35921

**Published:** 2023-03-14

**Authors:** Thomas Chirwa, Steven Malinga, Malelo Ilukena, Richard Bwalya, Chitalu Miriam Chama-Chiliba

**Affiliations:** 1 World Vision Zambia, Lusaka, Zambia,; 2 World Vision International, Mbabane, Eswatini,; 3 Institute of Economic and Social Research, Lusaka, Zambia

**Keywords:** Health care seeking, childhood illnesses, developing countries

## Abstract

**Introduction:**

mortality in under-five children remains a significant challenge in developing countries, including Zambia, where pneumonia and malaria account for twenty percent of under-five deaths. Poor health care seeking is one of the contributors to the high mortality rates. This study examined the predictors of health care seeking for acute respiratory infection (ARI) and fever among under-five children in Zambia.

**Methods:**

the study used a population based cross-sectional survey program evaluation dataset with sample size of 12,507 households from 28 districts. Binary logistic regression was used to examine the determinants of appropriate care seeking for ARI or fever, ARI, and fever.

**Results:**

the prevalence of fever or ARI in children under five was 22.9%, ARI 12.9%, and fever 13.4%. Educational status and non-participation in positive deviance hearth (PDH) were significant predictors in those with fever or ARI. Children whose household head had secondary education or higher were 4.5 times more likely to seek care than those whose household head did not have any education. Among those with ARI, educational status, women empowerment in decision-making and growth monitoring and promotion (GMP) were significant predictors while for fever only GMP was a significant predictor.

**Conclusion:**

over two thirds of caregivers sought care appropriately for fever or ARI. Only educational status and GMP were associated with more than one appropriate care seeking outcome. Through GMP services, policymakers can improve healthcare seeking behavior in children under five.

## Introduction

Globally, under-five mortality declined, with rates falling from 93 deaths per 1000 live births in 1990 to 37 in 2020 [1]. Despite the progress, the under-five mortality rates remain unacceptably high in sub-Saharan Africa (SSA), with most deaths being due to preventable causes [2]. In Zambia, the under-5 mortality rate has continued to decline steadily and fell from 75 deaths per 1,000 live births in 2013-14 to 61 deaths per 1,000 live births in 2018 [3]. Even though there are affordable and effective interventions for pneumonia, malaria, and diarrhea, they remain the leading causes of death in SSA [4]. In Zambia, one in every five deaths is attributable to pneumonia or malaria [5]. Delay in seeking care or failure to seek care is a key reason for the high rates of under-five death in resource-constrained settings [6]. Health care seeking behavior for childhood illness is not ideal in Zambia, and many children are not taken to a health care provider when they are sick [7]. In cases where appropriate care is sought, it is often late, thereby predisposing the children to poorer outcomes [8-10]. Most of the existing empirical literature on health care seeking behavior is from developing countries. Several studies have explored predictors of health care seeking for common childhood illnesses in resource-constrained settings; however, most of them were not nationally representative [6,11]. The situation in Zambia is not much different, with only a few studies [7,8,12] covering small areas of the country. The aim of the study was to identify the factors associated with care seeking for fever and acute respiratory infections (ARI) using data from eight out of the ten provinces in Zambia. Since this study covered nearly all provinces, the findings are more nationally representative.

## Methods

**Study design:**the study used a population-based cross-sectional design using data from a multi-sectoral end-of-program evaluation in Zambia.

**Study setting:**the setting for the study was 31 area programs (APs), predominantly in rural areas where World Vision Zambia implemented programs between 2017 and 2021. The programs covered interventions in livehoods, health and nutrition, water and sanitation, and education, covering 28 districts in eight (namely Northern, Lusaka, Eastern, Southern, North Western, Muchinga, Central and Western) of the ten Provinces in Zambia. Data was collected between April and June 2021.

**Data sources and target population:**the data were primarily collected for the evaluation of World Vision programs. Data were collected using an adapted version of World Vision's Caregiver Survey questionnaire for program evaluations [13]. The data were then cleaned and uploaded to a central server. The specific target population for this study was mothers and caregivers with children aged 0-59 months. Theinformation on fever and ARI were obtained based on the mother or caregiver's report.

**Study size:**the sample size was obtained using the World Vision sample size calculator, which is available from the Baseline field guide [14]. The statistical power to detect change was set at 0.84, with a design effect of 2, with the probability of committing a Type-1 error set at 1.96. The minimum sample size that was required in order to be able to detect statistically significant differences between baseline and evaluation proportions at the AP level was 358. Given that there were 31 APs, the overall total sample for the study was calculated as 11,098.

**Participants:**households interviewed were selected using two-stage cluster sampling. The first stage of sampling entailed random selection of villages from zones with probability proportion to size (PPS) used to ensure that larger villages and zones contributed more households than smaller ones. The zones included were selected by first listing all zones alphabetically and then using systematic random sampling to choose the ones to be included. In the second stage, households were selected using random walk method starting from the center of the villages selected.

**Data analysis:**the cleaned data from the server were exported into statistical package for the social sciences (SPSS) and analyzed. Weighting was done to correct for the equal sample sizes used at the AP level despite the differences in AP level populations. The sociodemographic variables were analyzed by running descriptive frequencies. Binary logistic regression models were used to determine the factors significantly associated with each of the three outcomes based on p<0.05. Missing data was excluded in each of the sub-analysis.

**Outcome variables:**the three main outcomes of interest were whether a child had fever or ARI, fever, and ARI.

Fever was defined as a child who had a history of fever in the two weeks preceding the survey.

**Acute respiratory infection**was defined as a child who had a history of an illness in the two weeks preceding the survey with cough and breathing faster than usual with short, rapid breaths or had difficulty breathing.

**Fever or acute respiratory infection**was a combined variable that included children who had either fever or ARI or both in the two weeks preceding the survey.

**Appropriate health care seeking**was defined as a child who had ARI, fever or ARI and sought care from a health facility (private or public), outreach clinic, community health worker, physician, or pharmacy. Obtaining health care from a shop, friend/relative, or traditional healer was not considered appropriate health care seeking.

**Predictor variables**the factors assessed included educational status, marital status, involvement in positive deviance hearth (PDH), province, total number of people in the household, attendance of growth monitoring and promotion (GMP), women empowerment in decision-making, and poverty probability index (PPI).

**Educational status**was defined based on the highest education attained by the household head.

**Marital status**was defined as whether the household head was either married that is married or cohabiting or unmarried that is single, divorced/separated or widow/widower.

**Involvement in positive deviance hearth**was defined as households where anyone participated in PDH in the 12 months preceding the survey. Positive deviance hearth, first described by Zeitlin *et al*.[15], is an intervention that combines two approaches; i) positive deviance is premised on the concept that solutions to community problems exist within communities and only need to be identified; ii) hearth, where community members and caregivers practice new feeding, hygiene, and health-seeking behaviors that have successfully prevented and rehabilitated children with malnutrition.

**Attendance of growth monitoring and promotion**was defined as a child who had been taken for GMP at least once in the six months preceding the survey.

**Women empowerment in decision making**was calculated based on Indikit's average household decision-making score which uses ten questions to characterize women's involvement in decision making and which has been described elsewhere [16]. An average household decision-making score of 0.67 or higher was taken as a household where a woman is empowered in decision-making.

**Poverty probability index**was calculated best on the Zambia 2015 PPI user guide published by innovations for poverty action [17]. The socio-demographic variables are presented using frequencies while the factors evaluated for association with each of the outcomes of interest (fever or ARI, fever and ARI) are presented using adjusted odds ratios and p values.

**Ethical considerations:**ethical approval for the program evaluation was obtained from the Zambia ERES Converge Ethics Committee (reference number 2021-April-008). During the survey, informed consent was obtained from respondents, and all information obtained was kept confidential. All personal identifiers were removed from the data before storing it securely and safely, with only authorized persons permitted access to it.

## Results

**Sociodemographic characteristics:**the total number of households in the study was 12,507, with an average size of 6.7. The vast majority (82.9%) of household heads were married, and more than half (50.5%) had primary as their highest level of education as shown in Table 1.

**Table 1:** characteristics of study households

Variable	Frequency	Percentage	Total (n)
**Educational status of household head**			12,507
None	1,088	8.7%	
Primary	6,272	50.1%	
Secondary and above	5,049	40.4%	
Missing	98	0.8	
**Marital status of household head**			12,507
Unmarried	2,139	17.1%	
Married	10,368	82.9%	
**Participation in PDH**			12,507
Yes	1,272	10.2%	
No	11,235	89.8%	
**Province**			12,507
Northern	2,934	23.5%	
Eastern	1,858	14.9%	
Lusaka	1,423	11.4%	
Southern	3,586	28.7%	
Central	659	5.3%	
North Western	877	7.0%	
Muchinga	737	5.9%	
Western	433	3.5%	
**Participation in GMP**			12,507
Yes	11,752	94.0%	
No	755	6.0%	
**Decision making index**			12,507
Women involved in decision making	5,748	71.4%	
Women not involved in decision making	2300	28.6%	
Missing	4,459	3.6%	

Average household size 6.7: average PP1 70.4; PDH: positive deviance hearth: GMP: growth monitoring and promotion

**Table 2:** multivariate logistic regression of factors associated with appropriate health care seeking for fever or acute respiratory infections, n=1.276

Variables	Adjusted odds ratio (95% CI)	P value
**Education status of household head**		
None	1.0	
Primary	2.46 (0.99-6.10)	0.053
Secondary and above	4.48 (1.55-12.95)	0.006*
**Marital status of household head**		
Married	1.0	
Unmarried	1.41 (0.44-4.53)	0.563
**Participation in positive deviance hearth**		
Yes	1.0	
No	2.30 (1.02-5.20)	0.044*
**Province**		
Western	1.0	
Northern	1.90 (0.61 – 5.91)	0.269
Eastern	2.36 (0.65-8.62)	0.194
Lusaka	1.12 (0.27 – 4.66)	0.876
Southern	1.50 (0.45 – 5.02)	0.515
Central	1.11 (0.32-3.84)	0.869
^a^North Western		0.997
Muchinga	4.52 (0.79-26.03)	0.091
Household size	1.11 (0.97 – 1.27)	0.135
**Participation in GMP**		
No	1.0	
Yes	2.02 (0.58-6.98)	0.268
**Decision making index**		
Women involved in decision making	1.0	
Women not involved in decision making	0.98 (0.51- 1.88)	0.954
Poverty index (PPI)	0.99 (0.98 -1.01)	0.445

a not provided as sample size was too small; *p<0.05 **p<0.01 ***p<0.001

**Childhood illness and care seeking:**the findings show that 2,867 (22.9%) children under five had ARI or fever in the two weeks preceding the survey, of which 2,132 (74.4%) were taken for care appropriately. There were 1,687 (13.4%) under-five children who had fever and 1,611 (12.9%) who had ARI. The proportion of caregivers who sought care appropriately for children with fever (79.4%) was better than those with ARI (68.8%).

**Factors associated with health care seeking for childhood illness (fever or ARI):**Table 2shows the binary logistic regression results of factors associated with appropriate health care seeking for fever or ARI. Caregivers whose household head had secondary education or higher were four and half times more likely to seek health care appropriately compared to those whose household head had no education (adjusted odds ratio (AOR) 4.48; 95%, confidence interval (CI) 1.55-12.95 p 0.006). Caregivers whose household head had primary education were two and a half times more likely to seek health care appropriately compared to those whose household head had no education (AOR 2.46; 95% CI 0.99-6.10, p 0.053). The study also found that participation in PDH was negatively associated with care seeking for fever or ARI. Caregivers whose households did not participate in PDH were about two times more likely to seek care appropriately than those who participated in PDH (AOR 2.30 95% CI 1.02-5.20), and this was marginally significant (p 0.044). Marital status, province, household size, participation in GMP, women's involvement in decision-making, and PPI were not associated with appropriate health care seeking for children with fever or ARI.

**Factors associated with appropriate health care seeking for ARI:**binary logistic regression results showed that educational status, participation in GMP, and women's participation in decision-making were the factors associated with appropriate health care seeking for ARI see Table 3. Children whose household head had primary education were twice as likely to seek appropriate care for ARI as those who did not have any education (AOR 2.02; 95% CI 1.12-3.63, p 0.019). There was a significant relationship between attending GMP and appropriate care seeking for ARI. Caregivers who attended GMP were almost three times more likely to seek health care appropriately than those who did not participate in GMP (AOR 2.81; 95% CI 1.42-5.60, p 0.003). Caregivers in households where women were involved in decision-making were twice as likely to seek care appropriately for ARI compared to those where women were not involved in decision-making (AOR 2.07; 95% CI 1.48-2.89, p<0.001). Marital status, participation in PDH, province, household size, and PPI did not predict care seeking for ARI.

**Table 3:** multivariate analysis of the factors associated with fever and acute respiratory infections

Variable	Care seeking for fever, n=925 adjusted odds ratio (95% CI)	p value	Care seeking for ARI, n=940, adjusted odds ratio (95% CI)	p value
**Education status of household head**				
None	1.0		1.0	
Primary	0.91 (0.47- 1.79)	0.791	2.02 (1.12 - 3.63)	0.019*
Secondary and above	1.52 (0.74 –-3.14)	0.256	1.59 (0.88 -2.89)	0.128
**Marital status**				
Married	1.0		1.0	
Unmarried	0.69 (0.39 – 1.23)	0.210	0.92 (0.56 – 1.52)	0.752
**Participation in PDH**				
Yes	1.0		1.0	
No	0.70 (0.39 – 1.27)	0.244	1.21 (0.75 – 1.95)	0.438
**Province**				
Western	1.0		1.0	
Northern	0.95 (0.51 – 1.77)	0.876	0.81 (0.42 – 1.55)	0.518
Eastern	1.74 (0.81 – 3.73)	0.154	1.56 (0.76 – 3.22)	0.227
Lusaka	0.74 (0.31 – 1.77)	0.498	0.74 (0.36 – 1.51)	0.404
Southern	0.55 (0.27 – 1.09)	0.087	1.10 (0.57 – 2.12)	0.778
Central	0.86 (0.40 – 1.84)	0.699	1.02 (0.50 – 2.08)	0.955
North Western	2.41 (0.92 – 6.32)	0.074	2.90 (0.70 – 12.08)	0.143
Muchinga	2.00 (0.84 – 4.78)	0.117	0.71 (0.33 – 1.55)	0.395
Household size	0.98 (0.92 – 1.04)	0.487	1.00 (0.96 – 1.05)	0.956
**Participation in GMP**				
No	1.0		1.0	
Yes	2.41 (1.17 – 4.97)	0.017*	2.81 (1.42 – 5.60)	0.003**
**Decision making index**				
Women are involved	1.0	;	1.0	
Women are not involved	0.91 (0.63 – H1.32)	0.634	2.07 (1.48 – 2.89)	<0.001***
**PPI**	1.00 (0.99 – 1.00)	0.322	1.00 (1.00 – 1.01)	0.885

GMP: growth monitoring and promotion; PPI: poverty probability index; PDH: positive deviance hearth; ARI: acute respiratory infections

**Factors associated with appropriate health care seeking for fever:**participation in GMP was the only significant predictor for care seeking for fever see Table 3. Caregivers in households that attended GMP were more than twice as likely to seek care appropriately compared to children in households that did not attend GMP (AOR 2.41; 95% CI 1.17-4.9 p 0.017). Education status, marital status, participation in PDH, household size, women's involvement in decision-making, and PPI were not associated with appropriate health care seeking for fever.

## Discussion

The study assessed the factors associated with appropriate health care seeking for children under five with ARI, fever, or a combination of either fever or ARI in Zambia. The study found that the prevalence of fever or ARI in children under five was 22.9%, fever 13.4%, and ARI 12.9%. The results for fever prevalence are similar to that of the Zambia Demographic Health Survey (ZDHS), which was 15.8%, but different for ARI prevalence which was 1.7%. The difference in the prevalence of ARI in this study and that of the ZDHS could be due to the differences in definitions between the two studies. In our study, ARI was defined as a child who had a history of a cough and breathing faster than usual with short rapid breaths/difficulty breathing. In contrast, the ZDHS study applied a stricter definition where fast breathing or difficulty in breathing had to be chest-related for it to count. In addition, ZDHS included both urban and rural areas, while our study covered primarily rural areas. The study found that appropriate health care seeking for children with fever was 79.4% compared to 68.8% for ARI. These findings are consistent with a similar study done in Zambia, which found that care seeking for children with diarrhea, ARI, or fever was 75.2% [12]. The level of care seeking found in our study is higher than in other resource-constrained settings such as Bangladesh(19.3%) [18], Nigeria (less than 32%) [19,20] and Ethiopia (less than 30%) [21]. The relatively high care seeking levels in Zambia could be due to the effective health promotion interventions that have been implemented in the country over the past two decades. Our findings show that amongst those who had either fever or ARI, education status and non-participation in PDH was associated with appropriate health care seeking. The poor health care seeking for caregivers who did not participate in PDH may be because participation in PDH is for underweight children who are more likely to be poor health care seekers.

Amongst those with fever, only attendance of GMP was found to be associated with appropriate health care seeking. Other studies show that determinants of health care seeking for children with fever are wealth, educational status, number of children under five, distance from the health facility, presence of both biological parents, caregivers perceived social norms, caregivers knowledge, location of residence and mother's decision-making score [18,22-24]. Early treatment for fever is positively correlated with geographical location and seasonality [7]. The factors related to care seeking for fever vary from study to study, which may be due to inter-country differences. Wealth and educational status are the more consistent factors associated with care seeking for fever in these studies [18,22-24]. Our study showed that participation in GMP, education status, and women's decision-making power were significant determinants of appropriate health care seeking in children with ARI. Other studies identified wealth, educational status, perception of illness severity, recognition of danger signs, distance from the health facility, place of residence, perceived treatment efficacy, positive view of the quality of health services, and availability of someone to care for family in cases of emergency [21,25-27]. Educational status and participation in GMP were the only factors associated with care seeking for at least two outcomes suggesting that they could be general predictors of care seeking among children under five.

Women's autonomy in making health care seeking decisions has gained significant importance in recent years [28]. Our findings showed that women's decision-making index was only associated with care seeking for ARI but not for fever, nor ARI or fever. The difference in women's decision-making power influence between ARI and fever care seeking is not apparent. A study by Akinyemi *et al*.in SSA found that the effects of women's decision-making index are greater at the community than at the individual level [29]. In Zambia, a study found that health care seeking was strongly associated with institutional delivery when measured at the cluster level but not at the individual level [30]. In this study, the effect of autonomy at the cluster level may have overshadowed the effect of autonomy at the individual level. Given that there are variations in cluster level autonomy in different parts of Zambia, it could have contributed to the differences observed in our findings which explored only individual-level effects and not cluster level autonomy in women's decision-making. Participation in GMP was associated with care seeking for both fever and ARI; this is likely because, during GMP sessions, mothers are counseled to seek care early and promptly in case their children have any illness. Since GMP is widely practiced in developing countries, it could be used to improve appropriate health care seeking in children under five. Poverty probability index was not significantly associated with appropriate care seeking for ARI or fever, ARI or fever which suggests that poverty per se does not necessarily affect care seeking. We postulate that for poverty to influence appropriate care seeking, it needs to interact with other factors to be a significant barrier. The findings of this study are more generalizable than that of previous studies since it covered 80% of the provinces whilst the other studies covered only small areas of the country [7,8,12]. However, the generalizability is limited to rural areas since the bulk of areas sampled were rural.

**Limitations:**the study used a data set that was not designed specifically to explore the study question, and thus, this limited the number of factors that could be explored. acute respiratory infection in this study was defined as a combination of cough and breathing faster than usual with short rapid breaths or having difficulty in breathing in the past two weeks. Acute respiratory infection is often used as a proxy for pneumonia; the drawback is that it tends to overestimate the prevalence of pneumonia [31,32]. However, in the absence of more robust and cost-effective tools to identify pneumonia, using a proxy remains the most commonly used approach and is employed by most large country surveys such as Demographic and Health surveys [33] and multiple indicator cluster surveys [34]. In addition, the main goal of this study was to explore the factors associated with care seeking rather than to estimate the prevalence of pneumonia.

## Conclusion

Over two thirds of children under five in Zambia with fever or ARI seek health care appropriately. The factors associated with health care seeking for ARI or fever, ARI and fever varied, with only education status and attendance in GMP being significant for at least two outcomes. The other factors associated with appropriate health care seeking were women's empowerment in decision-making and non-attendance in PDH. Policymakers could improve health care seeking behavior in children under five through expansion of uptake of GMP services.

### What is known about this topic

- *Health care seeking behavior for common childhood illnesses are influenced by both biological and social factors and varies from country to country;*
- *Health care seeking patterns can be improved through context specific modification of significant predictors of care seeking*.

### What this study adds

- *Over two-thirds of mothers in Zambia sought care appropriately for their children when ill with either fever or ARI, which is relatively high for a developing country;*
- *Health care seeking for children under-five could be improved through promotion of GMP which was found to be a predictor of health care seeking behavior*.
